# Polish attitudes towards unspecified kidney donation: a cross-sectional study

**DOI:** 10.1186/s12882-022-02767-x

**Published:** 2022-04-13

**Authors:** Paulina Kurleto, Lucyna Tomaszek, Irena Milaniak, Katrina A. Bramstedt

**Affiliations:** 1 Faculty of Medicine and Health Sciences, Andrzej Frycz Modrzewski University, ul. Gustawa Herlinga-Grudzińskiego 1, 30-705 Krakow, Poland; 2 John Paul II Hospital, Cracow, Poland; 3 Faculty of Health Sciences & Medicine, Bond University, 14 University Drive Gold Coast, Gold Coast, QLD 4226 Australia

**Keywords:** Poland, Organ donation, Kidney transplantation, Unspecified kidney donation, Legislation

## Abstract

**Background:**

Chronic kidney disease effects about 4.2 million people in Poland, yet Polish organ donation research is rare. In addition, compared to other countries in the world, Poland has a relatively low living donation rate. Still, little is known about how Poles would react to the possibility of living kidney donation to strangers. A study was conducted to examine public opinion about living kidney donation, as well as their knowledge about it, willingness to donate to a stranger, and support for a possible expansion of existing Polish organ donation legislation to include living donation to strangers.

**Methods:**

A self-report questionnaire, which included a socio-demographic datasheet (9 questions), 16 questions about attitudes towards living donation, and 1 question about knowledge concerning transplantation law was sent to the respondents from December 2020 – February 2021. Logistic regression was used to assess factors affecting the support of the legalization of unspecified kidney donation amongst the participants.

**Results:**

More than sixty percent (62.1) of respondents supported legalization of unspecified living kidney donation. Such legalization would be accepted by people who accept a choice of a family member to donate a kidney to a stranger (OR = 3.50; Cl 95%: 1.49 to 4.85), who think bone-marrow transplant is safe (OR = 2.65; Cl 95%: 1.80 to 3.91), recognize the benefit of carrying out tests before donating a kidney (OR = 2.56; Cl 95%: 1.79 to 3.69), would agree to receive a kidney from another person (OR = 2.24; Cl 95%: 2.53 to 3.13), or would agree to donate organs after death (OR = 2.06; Cl 95%: 1.45 to 2.95). However, support for unspecified living kidney donation would not be given by respondents fearing the risk of organ trafficking (OR = 0.54; Cl 95%: 0.38 to 0.79).

**Conclusions:**

In Poland there is strong support for legalization of unspecified living kidney donation. It is vital that future legislation define organ trafficking as a crime with serious punishment so that legal unspecified living kidney donation is not hindered.

**Trial registration:**

The study was registered with ClinicalTrials.gov (ID: NCT04789122) on 08/03/2021.

**Supplementary Information:**

The online version contains supplementary material available at 10.1186/s12882-022-02767-x.

## Background

Kidney transplantation is the preferred therapeutic option for patients with end-stage renal disease [1–3]. Numerous scientific evidence shows that organ transplantation, including kidney transplantation, improves quality of life and extends lifespan [4, 5]. According to the data from the Global Observatory on Donation and Transplantation, 100,097 kidneys were transplanted worldwide in 2019 [6]. Since 2020, the coronavirus pandemic has significantly impacted solid organ transplantation. Early during the pandemic, national and international transplant societies recommended suspending living kidney transplant programs in communities with widespread transmission to avoid exposing recipients considering their increased risk due to immunosuppression, and recommendations were made to reserve deceased-donor kidney transplantation for likely life-saving indications [7]. According data from Poltransplant (Polish Transplant Coordinating Centre Poltransplant), the number of deceased organ donors in Poland was 393 in 2020, which is a critical decrease compared to previous years [8, 9]. In 2020, a total of 1048 transplants were performed, and the average monthly number of patients waiting for a transplant was 1908. In 2020, 590 kidneys were transplanted from deceased donors. On average, 1,102 people waited for a kidney transplant each month [9].

Living kidney donation has been practiced in Poland since 1966 [10]. In 2020, however, only 31 living donor kidney transplants were performed. Historically, the maximum number of donors per year was 60 (2015). Compared to other countries in the world, Poland (38 million inhabitants) has a relatively low living donation rate. Notably, in 2020 in Spain (46 million inhabitants) 257 living donor transplants were performed, in Great Britain (67 million inhabitants)—558, in the Netherlands (17 ml inhabitants)—367, in Israel (8 ml inhabitants)—273, in Sweden (10 million inhabitants)—116, while in the USA (325 million inhabitants) 5.234 living kidney transplants were performed [8].

In Poland, in accordance with the *Act on the collection, storage and transplantation of cells, tissues and organs of July 1, 2005* [11], a living kidney donor can only be a person whose organ is donated to a relative in a straight line, siblings, an adopted person or spouse, and subject to Art. 13, for the benefit of another person, if it is justified by special personal reasons. Pursuant to Art. 13, the collection of cells, tissues or organs from a living donor for a person who is not a straight-line relative, sibling, an adopted person or spouse requires the consent of the district court having jurisdiction over the donor's place of residence or stay, issued in non-litigious proceedings, after hearing the applicant and after hearing the opinion of the Ethics Committee of the National Transplantation Council. According to Art. 13, paired donation transplantation is also allowed (Journal of Laws 2005, No. 169, item 1411) [11].

In some countries around the world, the pool of living donors has expanded beyond relatives and friends to include those unrelated to the recipient (e.g., donations to strangers; Good Samaritan donation; unspecified donation). In this article, we use the terminology proposed by the Ethical, Legal and Psychosocial Aspects of Transplantation (ELPAT) section of the European Society for Organ Transplantation (ESOT), which describes altruistic kidney donation to a stranger as “unspecified” [12]. Unspecified donors can donate their organ to a stranger on the waiting list or become a donor in paired exchange chains to facilitate matched pairs [13]. Donation to a stranger is legal in numerous countries, including the USA, Canada, Great Britain, Sweden, the Netherlands, Italy, Australia, and Israel [1, 2, 14–18].

Due to the significant decline in transplants in Poland and the ever-present transplant waiting list, a study was designed to examine Polish public opinion about living kidney donation, as well as their knowledge about it, willingness to donate to a stranger, and support for a possible expansion of the existing Polish law on "Collection, storage and transplantation of cells, tissues and organs" of July 1, 2005, to include unspecified living kidney donation (ULKD). To date, only one similar study was conducted in Poland in 2006 [19] on a large group of respondents (over 2,000 people).

## Methods

### Study design, setting, ethical considerations

A cross-sectional study was conducted from December 2020 – February 2021 among 960 randomly selected Polish adult residents, after obtaining the consent of the Bioethics Committee of the Kraków Academy of Andrzej Frycz Modrzewski (decision no. KBKA / 51 / O / 2020). Written consent was not required, as consent was presumed following the completion of a questionnaire by study participants. The guidelines of the Helsinki Declaration (World Medical Association, 2013) and STROBE (Strengthening the Reporting of Observational Studies in Epidemiology) [20] were followed, as well as The General Data Protection Regulation [21]. The study was registered with ClinicalTrials.gov (ID: NCT04789122).

### Participants

The study involved participants of both genders, over the age of 18 years, from all 16 Polish voivodeships (geographic regions within Poland similar to a province or state), who agreed to participate voluntarily and who completed the research questionnaire. Exclusion criteria were as follows: impaired verbal communication, inability to communicate in Polish, lack of Polish citizenship, age under 18 years. The participants did not receive an incentive or payment for participating in the research study.

### Variables

A diagnostic survey with the questionnaire technique was used in the study (Computer-Assisted Web Interview). The questionnaires were distributed in Polish via the Internet by the Statistical Research and Analysis Office (Rzeszów, Poland). Requests to complete the survey were sent to randomly selected Poles who agreed to receive various offers, including surveys. The register is a specialized database that is constantly updated. The database of respondents has been properly prepared for the needs of the research so as to reach the inhabitants of all voivodeships in Poland. The survey period was completed when the desired number of surveys was collected. It used a self-report questionnaire, which included a socio-demographic datasheet (8 questions), 17 questions about attitudes towards living donation (Cronbach’s alpha post-analysis = 0.67), and 1 question about knowledge concerning transplantation law. The questionnaire was created by the authors [PK, LT] but it was not validated (Polish and English versions of the questionnaire are provided as Supplement 1 and 2).

### Outcomes

The primary outcome assessed support for the legalization of ULKD in Poland.

The secondary outcomes revealed the factors influencing the support for the legalization of ULKD in Poland.

### Sample size

The structure of the sample was selected by stratified random sampling according to the representation in the population for gender, age and size of the place of residence. The minimum sample size to estimate the true population proportion with the required margin of error (3%) and confidence level (95%) for this study was 889 [22].

### Statistical methods

Categorical variables are represented by total number and percentage. The quantitative variable 'age' was presented as a median, upper and lower quartile, because the distribution of the variable differed from the normal distribution (Shapiro–Wilk test > 0.05).

Categorical variables between groups were compared with the chi-squared test. Logistic regression was used to assess the factors affecting the support of the legalization of ULKD in Poland (the dependent variable). The independent variables were sociodemographic variables and the attitudes of Poles. The construction of the multivariate model was based solely on independent variables, the significance level of which p in the univariate analysis was not greater than 0.05. The analysis was performed using the backward stepwise regression method, using a validation mechanism based on a v-fold cross-validation. Matching was assessed with the Hosmer-Lemeshov test (p > 0.05). Logistic regression results are shown with B, regression coefficient; SE, standard error; CI, confidence interval; OR, odds ratio. For two-sided tests, p values ​​below 5% were regarded as statistically significant. Statistical calculations were performed in STATISTICA v.13 (TIBCO Software Inc. (2017)).

## Results

### Participants’ characteristics

**Fig. 1:**
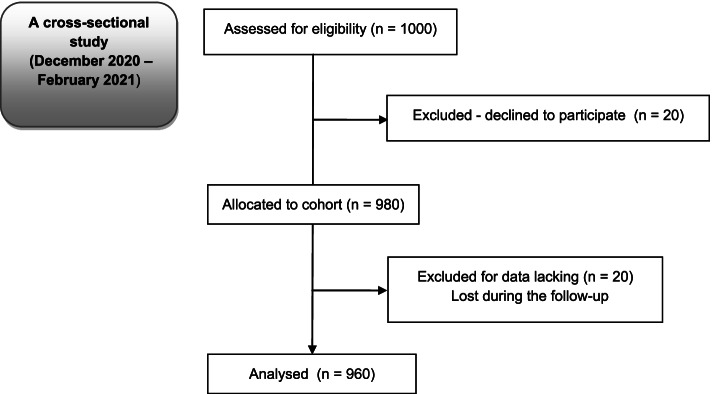
Flow diagram—study participants

Of the 1000 questionnaires distributed, 960 were completed, returned, and used for data extraction and analysis (96% response rate; Fig. 1). Participating adult Poles were aged 18–77 years (median 41) who represented each of the 16 voivodeships in Poland. Most were women (52.4%), married (65.1%) and had children (60.8%) and/or siblings (83.8%), and were Catholic (70.6%) city-dwellers (78.8%). Regarding education, 47.1% of participants had secondary, while 29.3% had higher education. Many participants were professionally active (42.1%). The sociodemographic data of the respondents are presented in Table 1.

**Table 1:** Sociodemographic characteristics of subjects (*N* = 960)

Variables	
Age (years)	41 [30; 60]
Gender	
Female	503 (52.4%)
Male	457 (47.6%)
Marital status	
Married	625 (65.1)
Divorced	43 (4.5)
Single	247 (25.7)
Widower/ Widow	45 (4.7)
Children – yes	584 (60.8)
Siblings – yes	805 (83.8)
Education	
University	281 (29.3)
High school	456 (47.5)
Vocational education	150 (15.6)
Primary school	73 (7.6)
Employment status	
Employed	404 (42.1)
Pensioner/Retired	285 (29.7)
Students	125 (13.0)
Unemployed	146 (15.2)
Place of residence	
City over 500,000 inhabitants	171 (17.8)
City from 100–500 inhabitants	234 (24.4)
City from 20–100 inhabitants	240 (25.0)
City up to 20,000 inhabitants	111 (11.6)
Village/rural	204 (21.2)
Religious self- identity	
Catholic	678 (70.6)
Not religious	282 (29.4)

Categorical data were reported as total number and percentage

Descriptive statistics are expressed as a median and upper and lower quartile

### Support for the legalization of ULKD in Poland depending upon sociodemographic factors

The highest support for the legalization of kidney donation in Poland to a stranger was recorded among the inhabitants of the West Pomeranian Voivodeship (81.5%), while the lowest (40.5%) in the Warmian-Masurian Voivodeship (*p* < 0.05). However, there was no statistically significant relationship between other sociodemographic factors (Table 2) and support for legalizing ULKD. This support (not statistically significant) was in the range of 57–66%, with the lowest support being declared by people who did not have any siblings, and the highest by rural residents.

**Table 2:** Support for legalization of unspecified kidney donation in Poland – sociodemographic factors

**Variables**	*Support for legalization*			
	*Yes*	*No*	*χ 2*	*p-value*
Gender				
Female (*n* = 503)	326 (64.8)	177 (35.2)	3.340	0.068
Male (*n* = 457)	270 (59.1)	187 (40.9)		
Marital status				
Married (*n* = 625)	386 (61.8)	239 (38.2)	0.080	0.778
Divorced/Single/ Widowhood (*n* = 335)	210 (62.7)	125 (37.3)		
Children				
Yes (*n* = 584)	356 (61.0)	228 (39.0)	0.801	0.371
No (*n* = 376)	240 (63.8)	136 (36.2)		
Siblings				
Yes (*n* = 805)	508 (63.1)	297 (36.9)	2.213	0.137
No (*n* = 155)	88 (56.8)	67 (43.2)		
Education				
University (*n* = 281)	172 (61.2)	109 (38.8)	0.129	0.720
High school/Vocational education/ Primary school (*n* = 679)	424 (62.4)	255 (37.6)		
Employment status				
Employed/ Pensioner/Retired (*n* = 689)	421 (61.1)	268 (38.9)	0.996	0.318
Students/Unemployed (*n* = 271)	175 (64.6)	96 (35.4)		
Place of residence				
City (*n* = 756)	461 (61.0)	295 (39.0)	1.844	0.175
Village/rural (*n* = 204)	135 (66.2)	69 (33.8)		
Religious self- identity				
Catholic (*n* = 678)	417 (61.5)	261 (38.5)	0.328	0.566
Not religious (*n* = 282)	179 (63.5)	103 (36.5)		

Data were reported as total number and percentage

### Support for the legalization of ULKD in Poland depending upon the knowledge and attitudes of Poles

Table 3 presents the results of the participants' responses regarding the legalization of ULKD in Poland. The majority of respondents believed that both blood donation (93.4%) and bone marrow donation (81%) were safe procedures for the donor. Nevertheless, only every third respondent has donated blood at least once (33%), and every tenth registered with a bone marrow donor bank (10.8%). People who had knowledge about the safety of blood donation (64.2% vs. 31.7%) and bone marrow (68.6% vs. 33.9%) more often declared their willingness to support the legalization of ULKD than those who did not have such knowledge (*p* < 001). A similar attitude was presented by the respondents who would agree to personal posthumous organ donation (68.8% vs. 40.9%; *p* < 001), providing consent for deceased donation from their relatives (69.8% vs. 47.9%; *p* < 001) and those respondents who did not see the need to ask the family of the deceased if while alive and in the presence of two witnesses, he or she expressed opposition to the removal of his organs after death (70.9% vs. 60.7%; *p* = 0.023).

**Table 3:** Support for legalization of unspecified kidney donation in Poland – knowledge and attitudes

**Variables**	*Support for legalization*			
	*Yes*	*No*	*χ 2*	*p-value*
Polish law allows the procurement and transplantation of kidney from living donors – knowledge				
Yes (*n* = 137)	91(66.4)	46 (33.6)	1.279	0.258
No (*n* = 823)	505 (61.4)	318 (38.6)		
View on blood donation				
Safe (*n* = 897)	576 (64.2)	321 (35.8)	26.361	< 0. 001
Unsafe (*n* = 63)	20 (31.7)	43 (68.3)		
Being a blood donor				
Yes (*n* = 317)	196 (61.8)	121 (38.2)	0.013	0.909
No (*n* = 643)	400 (62.2)	243 (37.8)		
View on bone-marrow transplant				
Safe (*n* = 780)	535 (68.6)	245 (31.4)	74.812	< 0.001
Unsafe (*n* = 180)	61 (33.9)	119 (66.1)		
Persons registered with the bone marrow donor bank				
Yes (*n* = 104)	73 (70.2)	523 (61.1)	3.258	0.071
No (*n* = 856)	31 (29.8)	333 (38.9)		
Consent to donate organs after death of participants				
Yes (*n* = 730)	502 (68.8)	228 (31.2)	57.824	< 0.001
No (*n* = 230)	94 (40.9)	136 (59.1)		
Consent to donate organs after death of a family member				
Yes (*n* = 622)	434 (69.8)	188 (30.2)	44.399	< 0.001
No (*n* = 338)	162 (47.9)	176 (52.1)		
Needed consent of the deceased for organ donation (given prior to death)				
Yes (*n* = 826)	501 (60.7)	325 (39.3)	5.138	0.023
No (*n* = 134)	95 (70.9)	39 (29.1)		
Knowing a person on dialysis, waiting for a transplant or has had a transplant				
Yes (*n* = 238)	156 (65.5)	82 (34.4)	1.612	0.204
No (*n* = 722)	440 (60.9)	282 (39.1)		
Consent to give one of your kidneys to another person				
Yes (*n* = 761)	503 (66.1)	258 (33.9)	25.127	< 0.001
No (*n* = 199)	93 (46.7)	106 (53.3)		
Consent to receive a kidney from another person				
Yes (*n* = 719)	489 (68.0)	230 (32.0)	42.753	< 0.001
No (*n* = 241)	107 (44.4)	134 (55.6)		
Organ trafficking risk				
Yes (*n* = 727)	431 (59.3)	296 (40.7)	9.966	0.002
No (*n* = 233)	165 (70.8)	68 (29.2)		
Selfless donation of a kidney to a stranger is something positive				
Yes (*n* = 538)	388 (72.1)	150 (27.9)	52.363	< 0.001
No (*n* = 422)	208 (49.3)	214 (50.7)		
Acceptance of a kidney donation by a family member to another family member				
Yes (*n* = 914)	571 (62.3)	343 (37.5)	1.228	0.267
No (*n* = 46)	25 (54.3)	21 (45.6)		
Acceptance of a kidney donation by the closest family member to a friend				
Yes (*n* = 880)	557 (63.3)	323 (36.7)	6.591	0.010
No (*n* = 80)	39 (48.8)	41 (51.3)		
Acceptance of the kidney donation by the closest family member to a stranger				
Yes (*n* = 702)	497 (70.8)	205 (29.2)	84.267	< 0.001
No (*n* = 258)	99 (38.4)	159 (61.6)		
Benefit of carrying out tests before donating the kidney				
Important for participants (*n* = 758)	523 (69.0)	235 (31.0)	73.155	< 0.001
Unimportant (*n* = 202)	73 (36.1)	129 (63.9)		

Data were reported as total number and percentage

Participants who would decide to voluntarily donate their kidneys were also more often in favor of legalizing ULKD (66.1% vs. 46.7%) or accepting kidney donation from other people (68.0% vs. 44.4%) compared to participants without such declarations (*p* < 0.001). Notably, a close emotional relationship with the recipient was one of the most important arguments for making a decision to donate a kidney. Specifically, respondents would most often donate a kidney to a child (70.8%), spouse (55.8%), siblings (49.8%) or to parents (48.2%); less often to a partner (27.1%) or friends (24.2%); and least often to a stranger (10.4%). In case of a need of a kidney transplant, spouses (46.8%) and siblings (46.9%) are the preferred donors. Notably, 31% of respondents indicated they would accept a ULKD for themselves.

An important factor motivating respondents to support the legalization of ULKD was the acceptance of the will of their closest family members to donate this organ to a partner / friend (91.7%) or a stranger (73.1%). People accepting the decisions of their relatives (63.3% and 70.8%, respectively) would more often support the act legalizing ULKD than people declaring no such acceptance (38.4% and 48.8%, respectively).

For the majority of the study participants (78.9%), the health and emotional benefits of living donor kidney transplantation, such as early detection of diseases in the donor due to many detailed pre-donation tests and regular post-transplant examinations, were important, strengthening the family bonds. The percentage of respondents wishing to support legalization of ULKD were approximately 33% lower in the group for which these benefits were not important.

Respondents who believed that ULKD could raise the risk of organ trafficking were also less likely to support legalizing this type of donation (59.3 vs. 70.8%; *p* = 0.002). Similarly, those who were not convinced that selfless donation to a stranger is a positive action were less likely to support ULKD legalization (49.3% vs. 72.1%; *p* < 0.001).

Variables such as: being a blood donor; being registered with the bone marrow donor bank; knowing a person on dialysis; waiting for a transplant or having received a transplant; acceptance for a member of the closest family to donate a kidney to another family member, did not affect the support for ULKD (*p* > 0.05).

### Factors related to support for the legalization of ULKD in Poland

The legalization of ULKD in Poland would be supported by 62.1% of Poles surveyed. In order to assess which variables described in Table 2 and Table 3 affected the support for the law legalizing ULKD, a logistic regression analysis was performed (Table 4). First, all variables were subjected to univariate analysis—variables whose significance level p was not greater than 0.05 were included in the construction of the multivariate model. Ultimately, the model included six explanatory variables for 29% of the model. The legalization of ULKD would be accepted by Poles who support ULKD by the closest family member (OR = 3.50; Cl 95%: 1.49 to 4.85), who think bone-marrow transplant is safe (OR = 2.65; Cl 95%: 1.80 to 3.91), recognize the benefit of carrying out tests before donating a kidney (OR = 2.56; Cl 95%: 1.79 to 3.69), would agree to receive a kidney from another person (OR = 2.24; Cl 95%: 2.53 to 3.13), or to donate organs after death (OR = 2.06; Cl 95%: 1.45 to 2.95). However, ULKD support would not be given by respondents fearing the risk of organ trafficking (OR = 0.54; Cl 95%: 0.38 to 0.79).

**Table 4:** Single-factor and multi-factor logistic regression model to support the legalization of kidney donation in Poland for a stranger

Variables	*B*	*SE (B)*	*Wald test*	*df*	*p*	*OR (Cl 95%)*
**Simple logistic regression**						
Blood donation is safe	1,35	0.28	23.34	1	< 0.001	3.85 (2.23 to 6.67)
Bone-marrow transplant is safe	1.45	0.17	68.31	1	< 0.001	4.26 (3.02 to 6.00)
Consent to give one of your kidneys to another person	0.80	0.16	24.47	1	< 0.001	2.22 (1.62 to 3.05)
Consent to receive a kidney from another person	0.98	0.15	41.33	1	< 0.001	2.63 (1.97 to 3.59)
Organ trafficking risk	-0.51	0.16	9.85	1	< 0.001	0.60 (0.45 to 0.82)
Selfless donation of a kidney to a stranger is something positive	0.98	0.14	51.17	1	< 0.001	2.66 (2.03 to 3.48)
Acceptance of the kidney donation by the closest family member to a friend	0.59	0.23	6.44	1	0.011	1.81 (1.14 to 2.87)
Consent to donate organs after death of participants	1.16	0.16	55.08	1	< 0.001	3.19 (2.35 to 4.33)
Consent to donate organs after death of a family member	0.92	0.14	43.41	1	< 0.001	2.51 (1.91 to 3.30)
Needed consent of the deceased for organ donation, which was given during his lifetime	0.46	0.20	5.08	1	0.024	1.58 (1.06 to 2.35)
**Logistic regression – multivariate model; R2 Nagelkerka = 0.29; Hosmer Lemeshow = 9.83, *p* = 0.08**						
Acceptance of the kidney donation by the closest family member to a stranger	1.25	0.17	57.13	1	< 0.001	3.50 (1.49 to 4.85)
Bone-marrow transplant is safe	0.97	0.20	24.26	1	< 0.001	2.65 (1.80 to 3.91)
Important benefit of carrying out tests before donating the kidney	0.93	0.19	24.96	1	< 0.001	2.56 (1.79 to 3.69)
Consent to receive a kidney from another person	0.81	0.17	22.28	1	< 0.001	2.24 (2.53 to 3.13)
Consent to donate organs after death of participants	0.72	0.18	15.95	1	< 0.001	2.06 (1.45 to 2.95)
Organ trafficking risk	-0.61	0.19	10.38	1	0.001	0.54 (0.38 to 0.79)

*B* regression coefficient, *SE* Standard error, *df* Degrees of freedom, *OR* Odds ratio, *CI* Confidence interval

## Discussion

The results of this study showed that over 60% of adult participants expressed their willingness to support the legalization of ULKD in Poland, and one in ten declared to voluntarily donate a kidney to a stranger. Their knowledge and attitudes regarding bone marrow transplantation or organ transplantation after death, organ transplantation from living donors, and the risk of organ trafficking were associated with support for the legalization of ULKD. Also, those viewing selfless donation of a kidney to a stranger as a positive act were more likely to support ULKD legalization compared to those who did not view donation in this manner. These data show that altruism is one of several variables to consider when approaching this topic for both Polish legislative change and ultimately UKLD promotion.

Compared to a similar, prior Polish study [19] our study indicates that support for ULKD is increasing. Namely, in 2006, Kowal et al. found only 0.2% of Poles supported ULKD, whereas 14 years later, we observed 10.4% supported ULKD. According to our study, nearly 80% (79.2%) of respondents would give a kidney to a close person. For comparison, in Denmark, 85.2% of respondents declared the readiness of living kidney donation to an identified person [23]. In Singapore, 48% of respondents would be willing to donate a living kidney, while 13.9% of those under 40, 17.4% of those aged between 40 and 60 and 31.4% of those over 60 years of age would consider donating to a stranger [24]. According research by Spital et al., 45% of respondents would donate a kidney to a stranger [25]. In Turkey, the percentage of people who would donate a kidney to a stranger is 26.8%, while 67% of respondents would give a kidney to a loved one [26].

Historical increases in the willingness to donate organs after death led to the hypothesis that support for legalizing ULDK donation may also increase over time. In Poland, attitudes towards organ transplantation have changed over the last two decades. Our research shows that 76% of Poles over 18 years of age would agree to posthumous organ donation, whereas in 2010 such a declaration was made by 53% [27].

As the logistic regression shows, legalization of kidney donation for a stranger would be accepted by Poles who support organ donation after death or who are convinced that bone marrow donation is safe. This potentially signals the value of donation education and knowledge in donation preferences. Accordingly, efforts to legalize ULKD would need to include educational endeavors that would explain ULKD in lay language across Poland. Benchmarking with educational materials used in other countries and adapting these to Polish culture should be performed so that the materials would be ready for use alongside a legislative change permitting ULKD.

In Poland, the percentage of living donation is low. A 2019 study on the attitude of Poles towards transplantation conducted by the Medical University of Szczecin shows that 58% of Poles do not trust the health care system [28]. According to 2010 data, 36% of Poles are afraid of manipulation on the human body [27]. Similarly, Nordfalk et al. found that 12.7% of Danish respondents have fears about not actually being dead during organ donation, and they also conclude that bodily integrity may be a concern for some people who do not support organ donation [23].

Moreover, 17.7% of respondents to our survey believe that a possibility of ULKD will greatly contribute to organ trafficking, and 58% believe that ULKD could promote organ trafficking.

People afraid of increasing the risk of organ trafficking will likely not approve ULKD legislative initiatives, thus it is vital that the legislation be clear that organ trafficking is a crime with serious punishment. As Poland is a predominately Catholic country (70.6% of our respondents declared to be Catholic), educational efforts should also include the Papal support for living and deceased organ donation [29]. Other potential concerns about ULKD such as donor screening and expenses, and the promotion of ULKD programs can be addressed by reflecting on best practices in high volume regions of the world [17, 29].

No statistically significant relationship was found between sociodemographic factors and support for legalizing ULKD in Poland (apart from living in certain voivodships), yet, there was a high level of support of the legalization of ULKD from those living in rural areas (66%). Perhaps rural Poles realize that the need for a kidney donor would be greater in their region (due to an inadequate supply of deceased donors or local living [donor] relatives. ULKD would provide a mechanism to clinically support the rural community who has end-stage renal disease, helping preserve the rural population. Notably, the West Pomeranian Voivodeship evidenced the strongest support for ULKD. This voivodeship is renowned for its wide array of health resorts and geothermal spas, as well as seven universities and one science-technology park – creating an environment that is clinically intellectual and innovative. There is an absence of such content in the Warmian-Masurian Voivodeship (lowest level of support for ULKD). The lowest level of ULKD support was noted amongst respondents who had no siblings. This may represent a generally pessimistic attitude about pursuit of transplant in the context of no family to rely on for support.

In 2010, only 27% of Poles surveyed declared knowledge of the law on organ transplantation [27]. In 2016, more than half of the respondents (51%) did not know what regulations regarding organ transplantation were in force in Poland. Our study found 30.6% of respondents do not know who, according to Polish law, can become a living kidney donor. More than half of the respondents (55.7%) said that only blood-relatives can donate kidneys to each other. This is a strong indicator that public education efforts must include clinical concepts that inform about the suitability for non-relatives to be effective “clinical matches” for people in need of kidney transplantation. People will not support ULKD if they think it is scientifically or clinically incorrect. Additionally, educational materials should include content that decries organ trafficking as both unethical and illegal.

Chronic kidney disease is the second most common chronic disease in Poland, after arterial hypertension, currently affecting about 4.2 million people. End-stage renal disease requiring dialysis or transplant impacts approximately 6,500 Poles yearly (approximately 170 people per million inhabitants), and the number of patients receiving dialysis is increasing by approximately 1.8% annually. It is estimated that in Poland, the number of dialysis patients may exceed 30,000 by 2030 [30]. Living kidney donation (including ULKD) is a viable clinical option to increase the number of kidney transplants and reduce dependence on costly and burdensome dialysis.

### Implications for clinical practice

The positive trend of Polish support for ULKD is encouraging. This sets the stage for legalization of ULKD, as well as a receptive population to educate about the process of ULKD, and arranging robust ULKD screening and support programs for these donors. The end result will benefit Polish patients with end-stage renal disease, as well as benefit the Polish health system (as transplantation is ultimately more cost-effective than dialysis) [31, 32].

Our study, amongst others [33, 34] has shown that whilst altruism is a significant driver for UKLD, it is not enough to facilitate these donations. There is a need for actions aimed at increasing knowledge and awareness regarding donation and transplantation. Tools include social media campaigns, educational programmes in schools, and popular science programmes targeting a wide audience. Content can include pre- and post-transplant stories, as well as donation stories in order to make concepts real rather than theoretical. Additionally, educational material should be balanced, including content pertaining to the risks (physical, psychological, financial) of living organ donation, and there should be follow up and support services for all living donors to ensure their welfare.

### Strengths and limitations

The strengths of the study is the large sample sizes. The structure of the sample was selected according to the representation in the population for gender, age and size of the place of residence. A minor study limitation is that the participant sample did not include other types of residents of Poland such as non-citizens (i.e., permanent residents); however, 99.2% of residents in Poland are Polish nationals [35]. Long-time residents of Poland might consider themselves “Polish” and are able to be both organ donors and recipients, yet their views regarding UKLD were not solicited as they are not eligible to vote (to change existing organ donation law). Also, there is a small risk of sampling bias with any mass crowdsourced survey cohort in that there is a risk of a subpopulation of frequent survey takers who know how to manipulate survey-taking (thus lowering effect sizes). This risk can be reduced by excluding participants who are known high-responders; however, we did not include this as an exclusion criterion. The questionnaire used within this study was not validated, and therefore the results must be interpreted with this in mind.

## Conclusion

Summarizing, over 60% of adult Poles would support the legalization of ULKD. Legalizing such donation would be accepted by people who believe that bone marrow donation are safe. It’s also supported by respondents who would accept a choice of a family member to donate a kidney to a stranger, and the ones that would decide to be deceased organ donors. ULKD would not be supported by people fearing the risk of organ trafficking, thus any change to the law permitting ULKD must have severe consequences for organ selling, human exploitation and trafficking.

## Supplementary Information

**Additional file 1.** Kwestionariusz ankiety własnej.

**Additional file 2.** Self-survey questionnaire.

## Data Availability

The data is stored by the authors of the study and may be made available at the request of the reader.

## Abbreviations

ULKD: Unspecified Living Kidney Donation
ELPAT: The European Platform on Ethical, Legal and Psychosocial Aspects of Organ Transplantation
ESOT: The European Society for Organ Transplantation
STROBE: Strengthening the Reporting of Observational Studies in Epidemiology
