# Supplementary material for: Polish attitudes towards unspecified kidney donation: a cross-sectional study
Source: BMC Nephrol. 2022 Apr 13;23:142. doi: 10.1186/s12882-022-02767-x (PMC9006497; doi:10.1186/s12882-022-02767-x)
Supplement: Supplementary file 1 — Additional file 1. Kwestionariusz ankiety własnej. [file 12882_2022_2767_MOESM1_ESM.docx]

**Kwestionariusz ankiety własnej**

1. **Płeć:**

- Kobieta
- Mężczyzna

1. **Wiek** [lata]:……………………
2. **Stan cywilny:**

- Wolny/a
- W związku (małżeńskim, nieformalnym)
- Rozwiedziony/Rozwiedziona
- Wdowiec/Wdowa

1. **Miejsce zamieszkania** - proszę napisać nazwę województwa………………………i zaznaczyć jedną z poniższych odpowiedzi:

- Miasto powyżej 500 tys. Mieszkańców
- Miasto od 100-500 tys. Mieszkańców
- Miasto od 20- 100 tys. Mieszkańców
- Miasto do 20 tys. Mieszkańców
- Wieś

1. **Wykształcenie:**

- Wyższe
- Średnie
- Zawodowe
- Gimnazjalne/ Podstawowe

1. **Obecna sytuacja zawodowa:**

- Zatrudniony (wykonywany zawód…………………………………………………)
- Bezrobotny
- Student/Uczeń
- Rencista/ Emeryt

1. **Czy posiada Pan/Pani dzieci?**

- Tak
- Nie

1. **Czy ma Pan/Pani rodzeństwo?**

- Tak
- Nie

1. **Czy jest Pan/Pani osobą wierzącą?**

- Tak (wyznanie……………………………………………………….......................)
- Nie-

1. **Czy kiedykolwiek w Pana/Pani życiu był/a Pan/Pani dawcą krwi?**

- Tak, jeden raz
- Tak, więcej niż raz
- Tak, jestem honorowym dawcą krwi
- Nie mogę z przyczyn zdrowotnych
- Nie, ale mam taki zamiar
- Nie, nie zamierzam

1. **Czy uważa Pan/Pani iż dawstwo krwi jest bezpieczne dla dawcy?**

- Zdecydowanie tak
- Raczej tak
- Nie wiem
- Raczej nie
- Zdecydowanie nie

1. **Czy jest Pan/Pani zarejestrowany/a w banku dawców szpiku:**

- Tak, byłem/am już dawcą szpiku
- Tak, ale nie byłem/am jeszcze dawcą szpiku
- Nie, ale planuję się zarejestrować
- Nie mogę z przyczyn zdrowotnych
- Nie, nigdy nie zamierzam

1. **Czy uważa Pan/Pani iż dawstwo szpiku jest bezpieczne dla dawcy?**

- Zdecydowanie tak
- Raczej tak
- Nie wiem
- Raczej nie
- Zdecydowanie nie

1. **Czy zna Pan/Pani osobę w swoim otoczeniu, która cierpi na niewydolność nerek i jest dializowana?** (można zaznaczyć kilka odpowiedzi)

- Tak - ja sam
- Tak - rodzic
- Tak - dziecko
- Tak - rodzeństwo
- Tak - małżonek/partner
- Tak - inny członek rodziny
- Tak - przyjaciel/ znajomy
- Nie znam

1. **Czy zna Pan/Pani osobę w swoim otoczeniu, która oczekuje, bądź jest po przeszczepie nerki?** (można zaznaczyć kilka odpowiedzi)

- Tak - ja sam
- Tak - rodzic
- Tak - dziecko
- Tak - rodzeństwo
- Tak - małżonek/partner
- Tak - inny członek rodziny
- Tak - przyjaciel/znajomy
- Nie znam

1. **Jak Pan/Pani osobę w swoim otoczeniu, która została dawcą nerki?**

- Tak (kim jest ta osoba?)………….
- Nie

1. **Czy zdecydowałby/łaby się Pan/Pani na dobrowolne oddanie nerki drugiemu człowiekowi?** (można zaznaczyć kilka odpowiedzi)

- Rodzicowi
- Dziecku
- Rodzeństwu
- Mężowi/Żonie
- Partnerowi/Partnerce
- Innemu członkowi rodziny
- Przyjacielowi
- Nieznajomej/anonimowej osobie
- Nie wiem
- Zdecydowanie nie

1. **Proszę wskazać osoby, od których zgodziłby/łaby się Pan/Pani przyjąć nerkę gdyby zaistniała taka konieczność** (można zaznaczyć kilka odpowiedzi)

- Rodzic
- Dziecko
- Rodzeństwo
- Mąż/Żona
- Partner/Partnerka
- Inny członek
- Przyjaciel
- Nieznajoma/anonimowa osoba
- Martwy dawca
- Trudno powiedzieć
- Od nikogo

1. **a Prawo dotyczące pobierania i przeszczepiania narządów w niektórych krajach na świecie dopuszcza altruistyczne (bezinteresowne) dawstwo nerki dla nieznajomego człowieka. Co Pan/Pani sądzi na ten temat:**

- Uważam to za heroiczny czyn, godny podziwu, też byłbym/byłabym gotowa to zrobić
- Uważam, że to wspaniały gest, ale sam nigdy bym tego nie zrobił
- Myślę, że to postawa godna naśladowania, mógłbym to rozważyć po dokładnym objaśnieniu konsekwencji dla mojego życia i zdrowia
- Uważam, że taki czyn to uczynienie siebie „kaleką” - nie można normalnie żyć z jedną nerką

19. **b Prawo dotyczące pobierania i przeszczepiania narządów w niektórych krajach na świecie dopuszcza altruistyczne (bezinteresowne) dawstwo nerki dla nieznajomego człowieka. Czy uważa Pan/Pani, że?**

- Ta procedura zdecydowanie ułatwi handel narządami
- Ta procedura może ułatwić handel narządami
- Nie mam zdania

1. **Kto w Pani/a opinii według Polskiego prawa może zostać żywym dawcą nerki** (można zaznaczyć kilka odpowiedzi):

- Osoby spokrewnione
- Małżonkowie
- Partnerzy, przyjaciele
- Dawcy w programie wymiany par
- Nie wiem

1. **Jakby Pan/Pani zareagował/a gdyby ktoś z najbliższych postanowił zostać dawcą nerki dla:**

|  | Zaakceptowałbym/ałabym to całkowicie | Zaakceptowałbym/ałabym tę decyzję ale z wieloma obawami | Przekonywałbym/  ałabym bliską osobę do zmiany decyzji | Nie byłbym w stanie zaakceptować tej decyzji |
| --- | --- | --- | --- | --- |
| 1. Członka rodziny |  |  |  |  |
| 1. Partnera/Przyjaciela |  |  |  |  |
| 1. Osoby nieznajomej |  |  |  |  |

1. **Z racji przeprowadzenia wielu szczegółowych badań przed pobraniem narządu i regularnych badań po przeszczepie, dawca ma szansę na wykrycie wielu chorób we wczesnym stadium rozwoju, co umożliwia wczesne leczenie, poprawia komfort życia w przyszłości i zwiększa szansę na dłuższe życie. Oddanie nerki bliskiej osobie przynosi korzyść psychologiczną, wzmacnia więzy rodzinne.** **Czy ważne są dla** **Pana/Pani te korzyści?**

- Zdecydowanie tak
- Raczej tak
- Nie mam zdania
- Raczej nie
- Zdecydowanie nie

1. **Czy poparłby/poparłaby Pan/Pani legalizację dawstwa nerki w Polsce dla osoby nieznajomej:**

- Zdecydowanie tak
- Raczej tak
- Nie mam zdania
- Raczej nie
- Zdecydowanie nie

1. **Czy zgodziłby/aby się Pan/Pani aby po śmierci zostały od Pana/Pani pobrane narządy?**

- Zdecydowanie tak
- Raczej tak
- Nie wiem
- Raczej nie
- Zdecydowanie nie

1. **Czy zgodziłby/aby się Pan/Pani gdyby od Pana/i bliskiego chciano pośmiertnie pobrać narządy?**

- Zdecydowanie tak
- Raczej tak
- Nie wiem
- Raczej nie
- Zdecydowanie nie

**26. Czy uważa Pan/Pani, że potrzebna jest zgoda zmarłego na pobranie narządów wyrażona za życia?**

- Tak
- Nie mam zdania
- Nie
