# Supplementary material for: Polish attitudes towards unspecified kidney donation: a cross-sectional study
Source: BMC Nephrol. 2022 Apr 13;23:142. doi: 10.1186/s12882-022-02767-x (PMC9006497; doi:10.1186/s12882-022-02767-x)
Supplement: Supplementary file 2 — Additional file 2. Self-survey questionnaire. [file 12882_2022_2767_MOESM2_ESM.docx]

**Self-survey questionnaire**

**1. Gender:**

• Woman

• Man

**2. Age [years]……..**

**3. Marital status:**

- Single
- Married/ Informal relationship
- Divorced
- Widowed

**4. Place of residence - please write the name of the voivodeship ………………………**

**and tick one of the following answers:**

- City over 500,000 Inhabitants
- City from 100,000 to 500,000 Inhabitants
- City from 20,000 to 100,000 Inhabitants
- City up to 20,000 Inhabitants
- Village/rural area

**5. Education**

- University
- High school
- Vocational school
- Primary school

**6. Employment status**

- Employed
- Pensioner/Retired
- Students
- Unemployed

**7. Do you have children?**

- Yes
- No

**8. Do you have siblings:**

- Yes
- No

**9. Are you religious?**

- Yes (religion ……………………………).
- No

**10. Have you ever donated blood in your life?**

- Yes, once
- Yes, more than once
- Yes, I am an honorary blood donor
- I can't due to health reasons
- No, but I am going to
- No, and I'm not going to

**11. Do you think blood donation is safe for the donor?**

- Definitely yes
- Rather yes
- I don’t know
- Rather not
- Definitely not

**12. Are you registered with the bone marrow donor bank?:**

- Yes, I have already been a bone marrow donor
- Yes, but I have not yet been a bone marrow donor
- No, but I plan to register
- I can’t due to health reasons
- No, and I’m not going to

**13. Do you think that bone marrow donation is safe for the donor?**

- Definitely yes
- Rather yes
- I don’t know
- Rather not
- Definitely not

**14. Do you know someone around you who suffers from kidney failure and is undergoing dialysis? (you can choose several answers)**

- Yes - myself
- Yes - parent
- Yes - child
- Yes - sibling
- Yes - wife/husband/partner
- Yes - another member of family
- Yes - friend
- No

**15. Do you know someone around you who is expecting or has had a kidney transplant? (you can choose several answers)**

- Yes - myself
- Yes - parents
- Yes - child
- Yes - sibling
- Yes - wife/husband/partner
- Yes - another member of family
- Yes - friend
- No

**16. Is there a person in your environment who became a kidney donor?**

- Yes (who is this person?)
- No

**17. Would you decide to voluntarily donate your kidney to another person? (you can choose several answers)**

- Parent
- Child
- Siblings
- Wife/Husband
- Partner
- Another member of family
- Friend
- Anonymously to a stranger
- I don’t know
- Definitely not

**18. Please indicate from whom you would agree to accept a kidney if necessary (you can choose several answers)**

- Parent
- Child
- Siblings
- Wife/Husband
- Partner
- Another member of family
- Friend
- Anonymous stranger
- I don’t know
- Deceased donor
- Hard to say
- From nobody

**19.a The law on organ donation and transplantation in some countries around the world allows altruistic (unrelated) kidney donation to a stranger. What do you think about this? [choice of one option]**

- I find it a heroic act, admirable, and I would also be ready to do it
- I think it's a great gesture, but I would never have done it myself
- I think this is an exemplary attitude, I might consider it after carefully understanding the consequences for my life and health
- I believe that doing so is crippling oneself - you can't live normally with one kidney
- I have no opinion

**19.b. The law on organ procurement and transplantation in some countries around the world allows altruistic (unrelated) kidney donation to a stranger. Do you think:**

- This procedure can greatly contribute to organ trafficking
- This procedure can contribute to organ trafficking
- This procedure does not affect the risk of organ trafficking

**20. Who, in your opinion, according to Polish law, can be a living kidney donor (you can choose several answers):**

- Related person
- A spouse
- Partner, friend
- Kidney exchange program participants
- I don’t know

**21. How would you react if someone close to you decided to donate a kidney to:**

|  | I would accept it completely | I would accept this decision but with many concerns | I would try to convince that person to change their mind | I would not be able to accept this decision |
| --- | --- | --- | --- | --- |
| 1. Family member |  |  |  |  |
| 1. Partner/friend |  |  |  |  |
| 1. To a stranger |  |  |  |  |

**22. Due to the fact that many detailed examinations are carried out before the donation of the organ and regular examinations after the transplant, the donor has a chance to detect many diseases at an early stage of development, which enables early treatment, improves quality of life in the future and increases the chance of a longer life. Giving a kidney to a loved one brings psychological benefits and strengthens family ties. Are these benefits important to you?**

- Definitely yes
- Rather yes
- I have no opinion
- Rather not
- Definitely not

**23. Would you support the legalization of kidney donation to a stranger in Poland:**

- Definitely yes
- Rather yes
- I have no opinion
- Rather not
- Definitely not

**24. Would you agree for your organs to be donated after your death?**

- Definitely yes
- Rather yes
- I have no opinion
- Rather not
- Definitely not

**25. Would you agree to donate your loved ones organs after their death?**

- Definitely yes
- Rather yes
- I have no opinion
- Rather not
- Definitely not

**26. Do you think it should be necessary to obtain a consent for posthumous organ removal during one’s lifetime?**

- Yes
- I have no opinion
- No
